# Effects of RANKL-Targeted Therapy in Immunity and Cancer

**DOI:** 10.3389/fonc.2013.00329

**Published:** 2014-01-07

**Authors:** Michael L. Cheng, Lawrence Fong

**Affiliations:** ^1^Division of Hematology and Oncology, Department of Medicine, Helen Diller Family Comprehensive Cancer Center, University of California San Francisco, San Francisco, CA, USA

**Keywords:** receptor activator of nuclear factor-kappa B, RANK ligand, dendritic cells, T-cell activation, immune tolerance, denosumab, prostate cancer, cancer immunology

## Abstract

The role of the receptor activator of nuclear factor-κB ligand (RANKL)/RANK system is well characterized within bone, where RANKL/RANK signaling mediates osteoclastogenesis and bone resorption. However, this system has also been shown to influence biologic processes beyond the skeletal system, including in the immune system and in cancer. RANKL/RANK signaling is important in lymph-node development, lymphocyte differentiation, dendritic cell survival, T-cell activation, and tolerance induction. The RANKL/RANK axis may also have direct, osteoclast-independent effects on tumor cells. Indeed, activity of the RANKL/RANK pathway in cancer cells has been correlated with tumor progression and advanced disease. Denosumab, a fully human monoclonal antibody against RANKL, inhibits osteoclastogenesis and is widely used not just for the treatment of osteoporosis, but for the prevention of skeletal-related events from bone metastases in solid malignancies such as breast and prostate cancer. The potential effects of denosumab on the immune system have been largely ignored. Nevertheless, with the emergence of immunotherapies for cancer, denosumab may impact the effectiveness of these therapies, especially if they are given in combination. In this article, we review the role of RANKL/RANK in bone, immunity, and cancer. Examining the potential effects of routine treatment with denosumab beyond the bone represents an important area of investigation.

## Introduction

The skeletal architecture is maintained through a complex remodeling process mediated by osteoblasts, responsible for bone formation, and osteoclasts, responsible for bone resorption. Osteoblasts are derived from mesenchymal stem cells, and their differentiation is induced by several specific transcription factors, including core-binding factor α1 (Cbfa1) and osterix (Osx), as well as by the bone morphogenic proteins BMP-2, BMP-4, and BMP-7. Mature osteoblasts synthesize and secrete type I collagen, the major structural protein in bone matrix. Osteoblasts also secrete non-collagen proteins including osteocalcin, osteopontin, and bone sialoprotein, as well as cytokines including IGF-1, IL-1, and IL-6 (1). In contrast, osteoclasts are hematopoietic in origin and derive from granulocyte-macrophage progenitor cells. Osteoclastogenesis requires stimulation by macrophage-colony stimulating factor (M-CSF) and the binding of receptor activator of nuclear factor-κB ligand (RANKL), expressed on osteoblasts, to RANK receptor on the osteoclast precursors. RANKL is a member of the tumor necrosis factor (TNF) family, as is also known as TNF-related activation-induced cytokine (TRANCE), osteoprotegerin ligand (OPGL), and osteoclast differentiation factor (ODF). Osteoclasts secrete proteases and hydrogen ions that dissolve and digest bone matrix, causing bone resorption. Osteoclast formation and function is stimulated by RANKL/RANK interaction, as well as by IL-1, IL-6, IL-11, transforming growth factor alpha (TGFα), TNFα, and TNFβ (2, 3). Osteoclasts are inhibited by multiple soluble factors, including OPG, an endogenous decoy receptor of RANKL secreted by osteoblasts and expressed in several other tissues, including lung, heart, and kidney. (4).

Tumor-derived factors including parathyroid hormone-releasing protein (PTHrP), prostaglandin E2 (PGE2), TNFα, and interleukins, have been demonstrated to enhance RANKL expression by osteoblasts and other bone stromal cells present, and diminish OPG expression (5). Some tumors, including prostate cancer, breast cancer, renal carcinoma, and multiple myeloma, produce RANKL which may directly contribute to osteoclastogenesis (6).

## Immunity and the RANKL/RANK System

Recent work has demonstrated that the RANKL/RANK system also serves an important role in the immune system, including in lymph-node development, lymphocyte differentiation, dendritic cell survival and T-cell activation, and tolerance induction. RANKL is essential in lymph-node organogenesis, as mice lacking this molecule develop without lymph nodes, but retain normal splenic architecture and Peyer’s patches (PP). Additionally, this work suggested that RANKL is important for the regulation of early T- and B-lymphocyte development, especially for CD25+CD44− thymocyte progression to CD25−CD44−, and for B220+CD43+CD25−pro-B-cell progression to B220+CD43−CD25+ pre-B cells (7). Furthermore, RANKL/RANK, along with CD40L/CD40, signaling is required for the development of medullary thymic epithelial cells (mTECs), which express the autoimmune regulator (AIRE) gene and mediate T-cell self-tolerance (8, 9).

**Figure 1:**
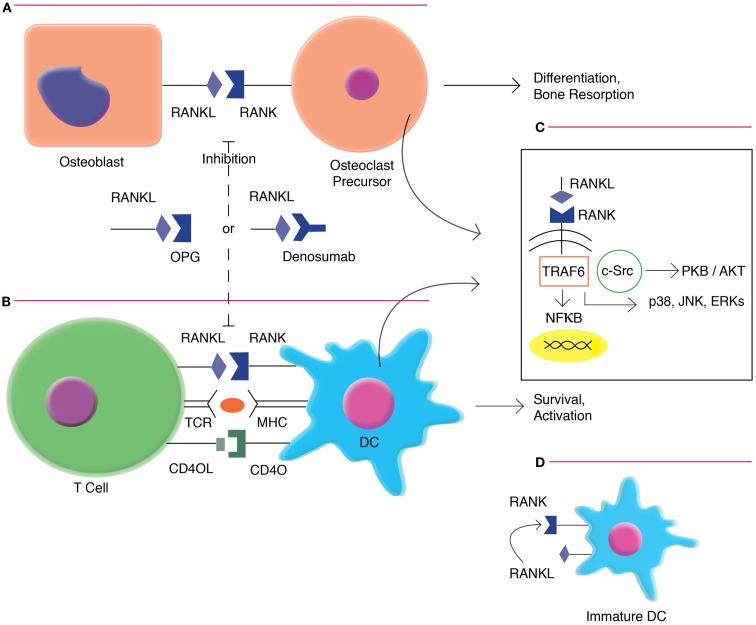
**RANKL/RANK signaling in osteoclast formation and DC activation**. **(A)**. RANKL/RANK interactions enhances osteoclast differentiation and bone resorption. **(B)** RANKL/RANK interactions also occur in the immune system, driving dendritic cell survival, and activation. **(C)** Signaling occurs via the recruitment of adaptor molecules, most importantly TNF receptor-associated factor 6 (TRAF6), which activates downstream signaling pathways, including that of nuclear factor-κB (NFκB) as well as mitogen-associated protein kinases (MAPK) such as p38, c-Jun N-terminal protein kinases (JNK), and the extracellular signal-regulated kinases (ERK). TRAF6 also complexes with c-Src to activate the antiapoptotic serine/threonine kinase AKT/PKB. **(D)** Immature interstitial DCs co-express both RANKL and RANK, and demonstrate autocrine stimulation. However, as these cells mature, they down-regulate RANKL and become dependent on exogenous factors.

Receptor activator of nuclear factor-κB ligand is primarily expressed on activated CD4+ and CD8+ T cells. RANKL is upregulated with T-cell receptor (TCR) stimulation, and may have direct effects on T cells via Jun N-terminal protein kinases (JNK) activation, including enhancing the cell’s own proliferation and function (10). RANKL interfaces with RANK, which is highly expressed on dendritic cells (DCs) (11). This interaction increases DC survival and enhances induction of T-cell responses. RANKL/RANK signaling (Figure 1) is mediated by the recruitment of adaptor molecules, most importantly TNF receptor-associated factor 6 (TRAF6) (12), which activates downstream signaling pathways, including that of nuclear factor-κB (NFκB) as well as mitogen-associated protein kinases (MAPK) such as p38, c-JNK, and the extracellular signal-regulated kinases (ERK) (13). TRAF6 complexes with c-Src to activate the antiapoptotic serine/threonine kinase AKT/PKB (14). RANK triggering also can enhance DC survival via induction of the antiapoptotic protein B-cell lymphoma-extra large (Bcl-xl) (15), which has been demonstrated to be critical to DC survival *in vivo* (16).

Receptor activator of nuclear factor-κB ligand induces DC expression of multiple activating cytokines, including IL-1, IL-6, IL-12, and IL-15 (17). Mature DCs pulsed with soluble RANK-L prior to immunization exhibited enhanced abundance and longevity in draining lymph nodes *in vivo*, as well as improved CD4+ T-cell priming to purified protein derivative (PPD) and ovalbumin (OVA) antigen (18). DCs transfected with recombinant adenovirus vectors demonstrated improved survival and maintenance of CD83 and CD86 surface markers with the addition of RANKL (19). OPG deficient mice demonstrate a twofold to fivefold greater capacity to stimulate T-cell proliferation, despite similar MHCII and CD86 levels, suggesting that the OPG’s function as a molecular brake on the RANKL/RANK system also occurs in the immune system (20). Transduction of DCs with RANKL-RANK, but not CD40L/CD40, pairs enhanced the expression of costimulatory signals and augmented corresponding effector and memory cytotoxic T-lymphocyte (CTL) responses toward a tumor-associated antigen (TAA) (21).

The CD40L/CD40 system has functional similarities to RANKL/RANK, and also enhances DC survival and antigen-specific T-cell responses. However, CD40L is only expressed on activated CD4+ cells, may act at an earlier phase during the immune response than RANKL (22). CD40L also influences T-cell dependent B-cell responses whereas RANKL does not (23, 24). RANKL/RANK has been demonstrated to enable CD4+ T-cell priming independent of CD40L/CD40. The two systems may cooperate, and the predominant pathway employed may be influenced by particular antigens. RANKL inhibition in wild-type mice resulted in modestly reduced CD4+ T-cell response to lymphocytic choriomeningitis virus (LCMV), and inhibition in CD40L-deficient mice resulted in the near absence of CD4+ T-cell proliferative response and IFN-gamma expression (25).

Autocrine RANKL/RANK signaling has also been demonstrated in the immune system. Immature interstitial DCs found in the dermis and liver co-express both RANKL and RANK, and demonstrate longevity most likely mediated in an autocrine fashion. However, as these cells mature, they down-regulate RANKL and become dependent on exogenous factors for activation and survival (26). RANKL has additionally been recently described to similarly upregulate monocyte/macrophage survival, effector function, and antigen presentation. This study also demonstrated that blockade of RANKL reduced death from lipopolysaccharide (LPS)-induced endotoxic shock in a murine model (27)

Receptor activator of nuclear factor-κB ligand/RANK signaling may also serve important inhibitory functions in the immune system. The RANKL/RANK system has been implicated in the induction of tolerance and may also modulate immunosuppression. RANKL treatment enhanced tolerance to oral OVA administration, and increased IL-10 mRNA expression in PP DCs, suggesting important differences between mucosal and peripheral DCs (28). RANKL-RANK signaling has been shown to be critical to CD4+CD25+ regulatory T cells (Treg) generation, but not effector function, in pancreatic lymph nodes. Blockade of the pathway in a CD8 + T-cell mediated model of type I diabetes, resulted in diminished Treg accumulation and rapid destruction of beta islet cells (29). RANKL expressed on keratinocytes in ultraviolet-inflamed skin interacts with epidermal DCs to increase the generation of local and systemic CD4+CD25+ Tregs (30). Interestingly, T-cells have also been shown to upregulate RANKL in response to tolerogenic signal, in the absence of RANK expression on DCs (31), and RANKL expression is also increased in CD8+ T cells undergoing deletion (32).

Negative regulation of RANKL/RANK by OPG also occurs in the immune system, and OPG appears to have direct DC effects. DCs in OPG knockout (KO) mice demonstrate prolonged survival as well as enhanced proinflammatory cytokine response to *Escherichia coli* LPS stimulation. Replacement of OPG to OPG KO cultures diminished survival and cytokine expression to wild-type levels (33). However, treatment with OPG has also been demonstrated not to affect cellular immunity with regard to contact hypersensitivity or *Mycobacterium* infection. This study also showed that OPG induced modest co-stimulation of T cells, as well as increased humoral responses, though the mechanism of this is unclear (34). OPG also acts as a decoy receptor for TNF-related apoptosis-inducing ligand (TRAIL) and inhibits TRAIL-mediated apoptosis. Interestingly, TRAIL in turn may inhibit the bone-protective function of OPG (35).

The clinical importance of immune changes driven by RANKL/RANK signaling is uncertain. RANKL and RANK KO mice do not demonstrate diminished DC or monocyte development, and the function of these cells remains intact, suggesting that the interaction may not be required for activation (7, 36). A study evaluating six individuals with autosomal recessive osteopetrosis secondary to mutations in TNFSF11, the gene encoding RANKL, did not identify significant differences from controls in the number of B and T-cell subsets, T-cell proliferation, or propensity to apoptosis. Lower levels of Th1 and Th2 cytokine expression following stimulation was seen in one individual. It is possible that these mutant RANKL proteins may retain immune, but not bone, functions (37).

## Cancer and the RANKL/RANK System

The RANKL/RANK system mediates important osteoclast-dependent pathologic processes in metastatic disease to bone. The “vicious cycle” of reciprocal feedback between tumor proliferation and bone breakdown is well described. The invasion of metastatic cells and the production of tumor-associated factors enhances bone resorption, which in turn causes the release of immobilized growth factors that further promote tumor proliferation. Multiple studies demonstrate diminished growth and increased apoptosis in skeletal tumor burden secondary to osteoclast inhibition with RANKL blockade. This concept has been shown in murine models of prostate cancer (38, 39), breast cancer (40), and multiple myeloma (41). However, studies indicate that the RANKL/RANK system may also have complex osteoclast-independent effects on malignancies. RANK-expressing tumors include prostate cancer, breast cancer, lung cancer, renal carcinoma, and melanoma. Importantly, a series evaluating multiple solid tumors did not demonstrate significant differences in RANK expression between primary tumors and corresponding bone metastases (42).

Increased expression of RANKL, RANK, and OPG is found in prostate carcinoma tissue whereas expression is low in normal cells. Furthermore, expression of all three proteins was positively correlated with clinicopathologic parameters indicating more aggressive and advanced disease, including Gleason score, PSA level, androgen receptor (AR) negativity, and presence of metastases (43). Additionally, RANKL has been shown in prostate cancer to activate IκB kinase α (IKKα), and inhibit expression of the metastasis suppressor Maspin, leading to progressive disease. Active IKKα was positively correlated with tumor infiltration of RANKL-expressing lymphocytes (44). RANKL expression has also been associated with epithelial-to-mesenchymal transition (EMT), a morphologic switch in which cancer cells upregulate mesenchymal-associated genes and assume an increasingly migratory and invasive phenotype with loss of cell-cell adhesion. Increased RANKL expression has been described in human prostate cancer EMT models, suggesting the protein may represent a novel marker of this transition (45).

The RANKL/RANK system is necessary for the development of lobulo-alveolar mammary structures and RANKL and RANK KO mice demonstrate an inability to lactate, as well as impaired proliferation of alveolar epithelium (46). In a mouse model of mammary tumorigenesis, gain-of-function in RANK signaling resulted in increased formation of pre-neoplasias and tumors, while inhibition of RANKL diminished tumorigenesis (47). Additionally, RANKL is induced by the progestin medroxyprogesterone acetate (MPA), and the axis has been implicated in the pathogenesis of progestin-mediated breast cancer. Inactivation of RANK or deletion of RANK resulted in diminished and delayed MPA-driven breast cancer. RANKL treatment decreased cell death in response to doxorubicin and irradiation (48). Tumor-infiltrating CD4+CD25+FoxP3+ Treg cells have been associated with more aggressive breast cancer phenotypes, and these cells have elucidated to be a major source of RANKL production. Pulmonary metastasis in a model of breast cancer driven by overexpression of the ErbB2 (c-Neu) proto-oncogene, was shown to be mediated by RANKL signaling both from Treg expression and exogenous administration (49). RANK overexpression has been demonstrated to directly induce EMT as well as stem-like phenotypes in both normal mammary epithelial cells and tumor cells. RANK mRNA is more highly expressed in ER and PR negative tumors, which generally have poorer prognosis, and is correlated with high pathologic grade and metastasis (50, 51).

The RANKL/RANK system has also been investigated in renal cell carcinoma (RCC), and may have different roles among the several histologic subtypes. Analysis of RCC tissues demonstrated that RANKL mRNA was significantly higher in clear cell RCCs as compared to papillary RCCs, chromophobe RCCs, or non-neoplastic tissues. RANK mRNA expression was increased and OPG expression was decreased in clear cell RCCs as compared to non-neoplastic tissue. The RANKL to OPG mRNA ratio was higher in clear cell RCCs than papillary RCCs and non-neoplastic tissue. Additionally, immunostaining for RANKL protein expression was significantly greater in clear cell RCCs than the other histologies. In a clear cell RCC cell line, Caki-1, RANKL transfection accelerated tumor migration fourfold and led to greater dispersion and involvement of tumor margins histologically as compared to control. OPG administration blocked this effect in a dose-dependent manner. Clinically, patients with RANKL- and RANK-high tumors were seen to have shorter disease-free survival, disease-specific survival, bone-metastasis-free survival. OPG-high tumors were correlated with longer disease-free survival, and no OPG-high tumor developed bone metastasis (52).

Receptor activator of nuclear factor-κB ligand-triggered tumor migration was demonstrated in a lung cancer model via upregulation of intercellular adhesion molecule-1 (ICAM-1) (53). Regression of metastatic lung adenocarcinoma driven by an anaplastic lymphoma kinase (ALK) translocation with denosumab treatment by has been described in a case report. Down-regulation of AKT/PKB, which is downstream of the ALK fusion product, secondary to the lack of PI3K activation in RANK inhibition has been postulated as a possible mechanism of this observation (54). RANKL expression has been found in a proportion of HCV- and HBV-driven hepatocellular carcinoma (HCC), and significantly correlated to the development of bone metastases (55). RANKL-expression is also found in multiple myeloma and chronic lymphocytic leukemia, and induces cytokines involved in disease pathogenesis, including TNF, IL-6, and IL-8. A novel Fc-engineered RANK-Fc fusion protein, but not denosumab, was shown to induce NK cell-mediated antitumor immunity against RANKL-expressing targets (56). RANKL, RANK, and OPG were variably expressed in tumors of the thyroid, including papillary carcinomas, medullary carcinomas, and macrovesicular adenomas (57).

The RANKL/RANK pathway is may exert important effects on the behavior of cancer cells within the bone microenvironment. RANKL expressed by osteoblasts and stromal cells in bone tissue may represent an important chemoattractant or “soil” factor that underlies preferential metastasis of certain tumors to the bone. RANKL stimulation of prostate cancer cells can induce multiple signaling pathways which stimulate cellular migration, chemotaxis, and invasion through collagen matrix (58). RANKL, RANK, and OPG expression was greater in bone metastases than lymph-node metastases in prostate cancer (43). Similarly, RANK-expressing breast and prostate cancer cell lines demonstrate upregulation of matrix metalloproteinase-1 (MMP-1) and subsequent migration and invasion upon RANKL administration (59). High RANK and low OPG expression in primary breast cancer tumors correlated with accelerated bone metastasis and shortened skeletal-disease-free survival (60). *In vivo* RANKL inhibition in malignant melanoma reduced metastasis to bone, but not other sites, and diminished morbidity. These findings were independent of osteoclast-mediated effects (61).

The role of OPG may be an important factor in several malignancies. OPG has been demonstrated to inhibit apoptosis in a range of tumor cells, likely through its action as the decoy receptor for TRAIL. OPG has been demonstrated to be a survival factor in prostate cancer (62) and well as breast cancer (63), and multiple myeloma (64). Increased serum OPG has also been correlated with poor prognosis in gastric carcinoma (65) and bladder carcinoma (66).

## Clinical Trial Experience with Denosumab

Denosumab is a fully human monoclonal antibody that avidly binds RANKL and blocks RANKL/RANK interaction and signaling. Unlike OPG, denosumab does not bind to TRAIL (67). Denosumab is FDA-approved for the prevention of skeletal-related events (SREs) from bone metastases in solid malignancies other than multiple myeloma (68), as well as for the treatment of osteoporosis in postmenopausal women and men at high risk for fracture (69). The agent, administered subcutaneously, is dosed at 120 mg every 4 weeks for the prevention of SREs, and dosed at 60 mg every 6 months for its osteoporosis and bone loss indications. The mean half-life of denosumab is 28 days.

A meta-analysis, including three phase III studies, evaluating denosumab in comparison to zoledronic acid for the reduction of SREs demonstrated significantly decreased incidence of SREs (risk ratio (RR) 0.84, 95% confidence interval (CI) 0.80–0.88), as well as delayed onset of first SRE (RR 0.83, 95% CI 0.75–0.90) and time to worsening of pain (RR 0.84, 95% CI 0.77–0.91). Denosumab, which is not renally excreted, had a lower incidence of renal toxicity, but had an increased risk of hypocalcemia. No differences were seen in the incidence of new cancers, infections, or osteonecrosis of the jaw (ONJ) (70). A recent phase III trial also demonstrated that monthly denosumab in non-metastatic castration-resistant prostate cancer significantly increased bone-metastasis-free survival and delayed time to first bone metastasis compared to placebo, suggesting another potential role for the therapy. No difference in infections was seen, however denosumab was associated with increased incidence of ONJ and hypocalcemia (71). A phase III study investigating adjuvant denosumab for the prevention of bone metastasis in early-stage breast cancer is ongoing (D-CARE, NCT01077154).

The FREEDOM trial evaluated twice yearly denosumab for the prevention of fractures in postmenopausal women with osteoporosis. The phase III trial demonstrated decreased incidence of vertebral fracture (RR 0.32, 95% CI 0.26–0.41), hip fracture [hazard ratio (HR) 0.60, 95% CI 0.37–0.97], and non-vertebral fracture (HR 0.80, 95% CI 0.67–0.95) as compared with placebo. The incidence of cancer or infection was not increased in the treatment group, and there were no reported cases of hypocalcemia or ONJ with denosumab (72). Results from the first 2 years of the FREEDOM extension did not demonstrate a trend toward increased incidence of malignancy or infection over time. ONJ was reported in two patients in the cross-over denosumab group of the extension trial (73). Denosumab was also studied for the prevention of osteoporosis in men with non-metastatic prostate cancer receiving androgen-deprivation therapy, which is associated with bone loss and fractures. A phase III study demonstrated significantly increased bone mineral density at all measured sites and decreased incidence of new vertebral fractures with treatment. Adverse events were comparable between the denosumab and placebo groups. Infection-related serious adverse events were seen in 4.6% of patients receiving placebo, and 5.9% of patients receiving denosumab. No change in PSA levels over time were detected, and there were no cases of ONJ (74).

## Conclusion

The role of RANKL/RANK in immunity is complex, and evidence suggests that this system has multiple divergent effects, both in the generation of active immune responses, as well as in the induction of tolerance (Table 1). RANKL/RANK may have differential roles among particular populations of DCs and other immune cells. This system has been also been shown to influence disease processes outside of the skeletal system, including in cancer. While the osteoclast-dependent effects of RANKL/RANK signaling in bone metastases are well described, recent data has shown that RANKL/RANK signaling may have osteoclast-independent, direct tumor effects. The system has been studied in a range of malignancies, and RANKL/RANK activity has largely demonstrated a positive correlation with tumor progression and advanced disease.

**Table 1:** **Divergent effects of RANKL/RANK signaling on the immune system**.

Enhancement of immunity	Inhibition of immunity
Regulation of T- and B-lymphocyte development	Development of medullary thymic epithelial cells (mTECs), which mediate T-cell self-tolerance
Lymph-node organogenesis	Enhanced tolerance in Peyer’s Patch DCs
Increased DC survival, cytokine expression, and migration	Generation of regulatory T cells (Tregs)
Enhanced induction of T-cell responses	Induction of T-cell tolerance and deletion

Denosumab is routinely employed in clinical practice for the prevention of SREs in cancer and fractures in osteoporosis. No change in the rates of infection or new cancers was seen in clinical trials with denosumab, and long-term surveillance is ongoing (75). Treatment is associated with a significant risk of ONJ, the etiology of which is unclear (76). Treatment-induced effects on immunity and/or inflammation could play a role in this disease. This treatment related side effect is also seen with bisphosphonates, which are also known to have significant immunomodulatory effects beyond their effects on bone (77, 78). Otherwise, little clinical evidence exists to support significant global immune dysregulation due to RANKL inhibition. Also, there is evidence that suggests the presence of redundant pathways may limit consequential immune effects of denosumab administration (79). The present experimental evidence primarily suggests that RANKL/RANK signaling potentially mediates negative outcomes in cancer. While there is some evidence to suggest that OPG promotes tumor antiapoptosis, this is likely mediated by its inhibition of TRAIL, which is not a property shared by denosumab.

However, our understanding of the role of the RANKL/RANK pathway in cancer remains limited, and represents an important area of investigation. While denosumab may induce divergent effects of the immune system, these may occur at different times. For example, with initiation of denosumab, a reduction of mTECs or Tregs might transiently enhance anti-tumor immunity. These changes might be counterbalanced by the effects of denosumab on dendritic cell activation and tolerance over time, especially in cancer patients, who receive the agent at a higher dose and frequency. Further investigation may be helpful to assess whether the axis has positive or negative effects on anti-tumor immunity, especially in prostate cancer and melanoma, where FDA-approved immunotherapies are available. Immune-based therapies serve an increasingly important role in the management of solid malignancies, and include sipuleucel-T, an autologous dendritic cell vaccine against the prostatic acid phosphatase (PAP) antigen, as well as ipilimumab, a monoclonal antibody against CTL associated antigen 4 (CTLA-4). Patients may be inadvertently combining these treatments, the effect of which is unclear. Sequencing of denosumab with these immunotherapies could also potentially affect their immunogenicity. The RANKL/RANK axis may also be modified by other co-administered medications, and this represents an important area of investigation (80). Additionally, monitoring of the RANKL/RANK axis may potentially serve an important prognostic or diagnostic role in certain cancers.

## Author Contributions

Michael L. Cheng and Lawrence Fong contributed to literature review, manuscript writing, and editing.

## Conflict of Interest Statement

Dr. Lawrence Fong receives research support from Dendreon Corporation. Dr. Michael L. Cheng reports no conflict of interest.
